# ECCCOing the call for emergency and critical care training in low middle-income countries

**DOI:** 10.1186/s13054-019-2532-4

**Published:** 2019-07-05

**Authors:** B. Jason Brotherton, Pete Halestrap, Evelyn Mbugua, Hannah Gitura, David Aliba, Jonathan E. Matson, Burton W. Lee

**Affiliations:** 1 Kijabe Hospital, PO Box 20, Kijabe, 00220 Kenya; 2 Division of Pulmonary, Allergy & Critical Care Medicine, University of PIttsburgh School of Medicine, Pittsburgh, PA USA

Dear Editor,

We would like to express our keen interest in the article by Schell et al. “The global need for essential emergency and critical care” [1]. We agree that high-quality, cost-effective emergency and critical care should be part of any universal healthcare initiative.

In that regard, we would like to point out that Kijabe Hospital, a 350-bed tertiary referral hospital in central Kenya, has been training clinical officers in a nationally approved Emergency and Critical Care Clinical Officer (ECCCO) higher diploma program since 2015. Through our 18-month program, we have so far trained 16 clinical officers to effectively manage “*any immediately life-threatening, reversible condition*” [1]. An additional 20 are due to graduate by 2021. The program started with expatriate and Kenyan national physicians who collaborated to design the curriculum and establish the program. Now, many of our graduates serve as the primary teaching faculty at Kijabe as well as at other sites, promising sustainability and propagation of the program.

Utilising didactic and bedside teaching, our learners walk through a rigorous curriculum of evaluating, diagnosing and managing common and pressing conditions seen in our resource-constrained context. Additionally, technical skills of airway management, ventilator management and point of care ultrasound (POCUS) are taught.

Graduates, working in government and faith-based hospitals throughout Kenya, are at times the only clinician with advanced clinical skills to care for critically ill patients. At Kijabe, graduates function alongside other clinicians in the emergency department, ICU and triaging those on the wards that may be deteriorating. The impact of this has been notable across all areas of our hospital. Focusing on our emergency department, the mortality rate of 0.5% is a quarter of that published in other tertiary hospitals in Kenya [2].

Emergency and critical care is an exploding area in resource-limited settings with tremendous opportunity. It is our hope that as this program and other similar ones continue to grow across the region, an impact can be made on the estimated 45% of deaths which might be prevented by timely emergency care [3].

Clinical care, teaching and research are key in understanding what works and what is sustainable. To that end, mutually beneficial collaboration within resource-limited and from high-resource communities willing to understand the realities of local conditions is of utmost importance.

We would invite anyone wanting to gain a greater understanding of our program to email or visit us at their convenience.

## Data Availability

Not applicable
